# Sijunzi decoction granules in the prevention and treatment of recurrence of colorectal adenoma: Study protocol for a multicenter, randomized, double-blind, placebo-controlled trial

**DOI:** 10.3389/fphar.2023.1175811

**Published:** 2023-04-05

**Authors:** Wenjing Ni, Tao Liu, Yujing Liu, Lu Lu, Bingduo Zhou, Yancheng Dai, Hang Zhao, Hanchen Xu, Guang Ji

**Affiliations:** ^1^ Institute of Digestive Diseases, Longhua Hospital, Shanghai University of Traditional Chinese Medicine, Shanghai, China; ^2^ Shanghai Frontier Research Center of Disease and Syndrome Biology of Inflammatory Cancer Transformation, Shanghai, China; ^3^ Department of Gastroenterology, Yueyang Hospital of Integrated Traditional Chinese and Western Medicine, Shanghai University of Traditional Chinese Medicine, Shanghai, China; ^4^ Department of Gastroenterology, Shanghai Traditional Chinese Medicine-Integrated Hospital, Shanghai University of Traditional Chinese Medicine, Shanghai, China; ^5^ Department of Gastroenterology, Shanghai Municipal Hospital of Traditional Chinese Medicine, Shanghai University of Traditional Chinese Medicine, Shanghai, China

**Keywords:** traditional Chinese medicine, Sijunzi decoction, randomized controlled trial, colorectal adenoma, spleen deficiency syndrome

## Abstract

**Background:** The recurrence of colorectal adenomas (CRAs) after endoscopy predisposes patients to a risk of colorectal cancer. Guided by the traditional Chinese medicine (TCM), patients with colorectal diseases usually manifest with spleen deficiency syndrome (SDS) and are treated with Sijunzi decoction (SJZD). Therefore, this trial aims to explore the efficacy and safety of SJZD in the prevention and treatment of CRAs recurrence.

**Methods:** SJZD on prevention and treatment of CRAs recurrence after resection: a multicenter, randomized, double-blind, placebo-controlled trial was designed. Patients who undergo polypectomy of CRAs will be recruited and randomized into a SJZD group and a placebo group in a 1:1 ratio. The intervention phase will be 12 months. The follow-up period will last 24 months. The primary outcome is the CRA recurrence rate after intervention. The secondary outcomes include the CRA recurrence rate at the second year post-polypectomy, the pathological type of adenoma and the alterations in SDS scores after intervention.

**Discussion:** Previous clinical practice has observed the sound effect of SJZD in the context of gastrointestinal diseases. A number of experiments have also validated the active components in SJZD. This trial aims to provide tangible evidence for the usage of SJZD, hoping to reduce the recurrence of CRAs.

## Introduction

Colorectal adenoma (CRA), a common gastrointestinal disease, is widely recognized as a major precursor of colorectal cancer and plays an important role in the adenoma–carcinoma sequence in the carcinogenic pathway (Keum and Giovannucci, 2019). Endoscopy is currently routinely implemented in the clinic for early detection, periodic surveillance and prompt treatment of adenoma. Notably, the worry that CRA will recur after removal has been described. A previous meta-analysis reported that the local recurrence rate of adenoma after endoscopic mucosal resection was 88% during the first follow-up colonoscopy and increased to 91% at 6 months (Belderbos et al., 2014). Although advancements in endoscopic technologies and improved skills have relatively lowered the recurrence rate, some risk factors related to recurrence and repeated endoscopic resections still exist (Moss et al., 2015), imposing challenges in the diagnosis of colorectal cancer in the early stage. It is urgent to determine how to prevent and treat CRA recurrence.

Guided by traditional Chinese medicine (TCM), patients with colorectal diseases usually manifest with spleen deficiency syndrome (SDS) (Zhao et al., 2009)^,^ (Zhu et al., 2013), a comprehensive disease mainly referring to gastrointestinal symptoms, such as anorexia and diarrhea, and systemic symptoms, including fatigue, perspiration and fear of cold. Researchers found that spleen deficiency with dampness excess syndrome had the strongest correlation with CRA patients (Chen et al., 2021). To better diagnose this syndrome, a patient-reported outcome (PRO) scale for spleen deficiency has been successfully developed. It contains ten symptoms, each of which has its own weighting factor (Table 1) (Dai et al., 2022). Patients are diagnosed with SDS when the total score of all symptoms is greater than 20 (Dai et al., 2022). Our previous clinical study proved the feasibility of this rating scale (Dai et al., 2019a).

**TABLE 1:** Spleen deficiency rating scale.

Questions	Scoring weight (%)
Do you feel tired and lazy to speak?	20
Do you sweat easily?	5
Do you feel tasteless?	10
Are your stools thin and shapeless?	20
Do you drool a lot?	5
Do you have bleeding gums?	5
Do you feel your hands and feet not warm?	10
Do you suffer from insomnia?	5
Do you feel easy to catch a cold?	10
Do you have any abnormal changes in your eating habits recently, such as decreased appetite and abdominal distension after eating?	10

To counter SDS, our ancestors formulated Sijunzi decoction (SJZD). Since it first appeared in the book “*TaipingHuiminHejiJufang*” in the Song dynasty, SJZD has become a classical prescription in China. It comprises four ingredients: Radix ginseng (Renshen), Rhizoma Atractylodis macrocephalae (Baizhu), Poria (Fulin) and Radix Glycyrrhizae Preparata (Gancao). Chinese researchers have identified that after SJZD intervention, small intestinal peristalsis improved, and abnormal metabolic profiles returned to healthy conditions in an SDS rat model (Zhao et al., 2020). Shi et al. (2019) reported that polysaccharides, an essential extract of SJZD, were conducive to intestinal mucosal restoration, indicating the irreplaceable role of SJZD in treating gastrointestinal diseases. However, few studies have explored the prophylactic effect of SJZD on the recurrence of CRA. Therefore, a randomized, double-blinded, placebo-controlled, and multicenter clinical trial has been designed to investigate the efficacy and safety of SJZD granules in the treatment of patients with SDS after CRA polypectomy.

## Methods and analysis

### Study design and setting

**FIGURE 1:**
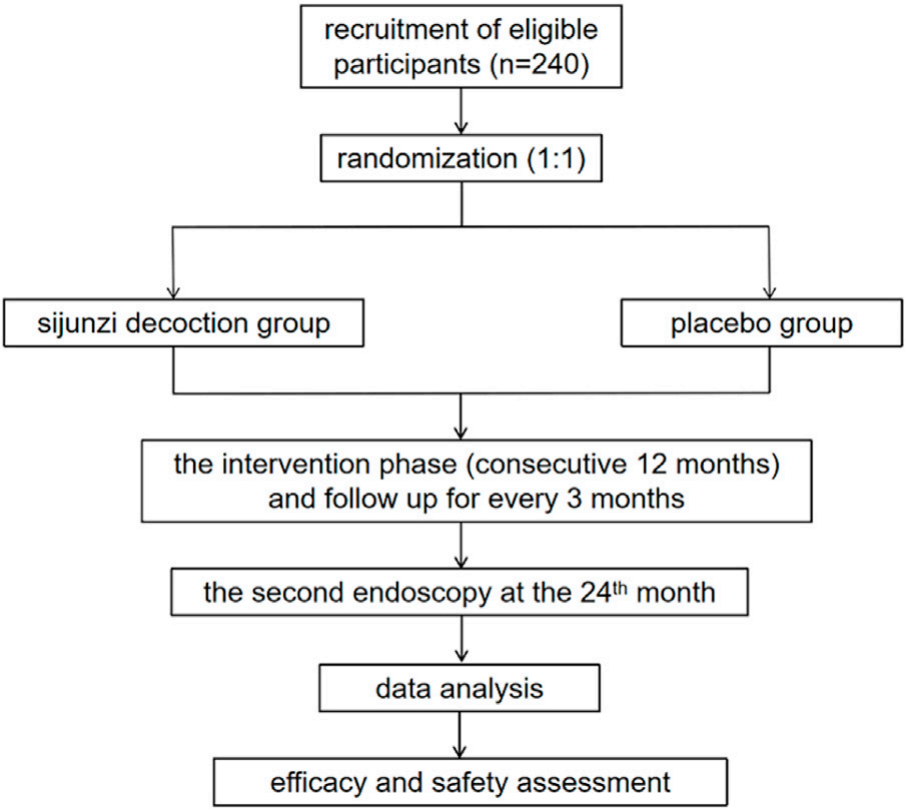
The flowchart of the trial.

The protocol was approved by the Medical Ethics Committee of Longhua Hospital Affiliated to Shanghai University of TCM (approval number: 2022LCSY031) and was registered in the Chinese Clinical Trial Registry (ChiCTR2200064391) on 6 Oct 2022 (http://www.chictr.org.cn/listbycreater.aspx). The flowchart of this trial is shown in Figure 1.

This is a multicenter, double-blinded, randomized controlled trial (RCT) of two parallel groups. A total of 240 cases will be recruited by recruitment advertisements and introductions from clinicians at outpatient or inpatient departments. Patients who meet the inclusion criteria will be informed of the possibility of participating in this study at the outpatient clinic or at admission. A face-to-face interview will be arranged to inform the patients about the purpose, interventions, examinations and follow-up procedures of this study. After informed consent acquisition, patients will be randomized to the SJZD group or placebo group at a ratio of 1:1. An intervention phase with a 12-month treatment with SJZD granules or placebo will be implemented for recruited patients. Endoscopy will be conducted at the end of the intervention phase and 1 year later. The duration of follow-up is 24 months with 5 visits, and the schedule details are listed in Table 2.

**TABLE 2:** Schedule of trial.

	Period					
Study phase	Enrollment	Visit 1	Visit 2	Visit 3	Visit 4	Visit 5
		3 months of interventions	6 months of interventions	9 months of interventions	12 months of interventions	The second year after intervention
Informed consent form	**√**					
Demographic information	**√**					
Inclusion and exclusion criteria	**√**					
General information	**√**					
Vital signs	**√**	**√**	**√**	**√**	**√**	**√**
Spleen deficiency rating scale	**√**	√	√	√	√	√
Endoscopy	**√**				√	√
Histological tests*	**√**				√	√
Routine blood tests	**√**	√	√	√	√	
Routine urine test	**√**	√	√	√	√	
Routine stool test	**√**	√	√	√	√	
Liver function	**√**	√	√	√	√	
Renal function	**√**	√	√	√	√	
Electrocardiograph	**√**	√	√	√	√	
Serum and stool samples collections	**√**				√	√
Adenomatous tissue collections*	**√**				√	√
Adverse events		√	√	√	√	√
Release drugs/placebo	**√**	√	√	√		
Retrieve empty packages			√	√	√	
Drug combination	**√**	√	√	√	√	√

Notes: Patients who are detected to find recurrence of colorectal adenoma and meet the criteria of resection are undergone endoscopy. Adenomatous tissue will be collected according to the relevant regulations and histology will be confirmed by pathological experts.

### Objectives

The major objective is to assess the efficacy and safety of SJZD granules in the prevention and treatment of CRA post-polypectomy.

### Participants

Patients will be recruited from gastrointestinal endoscopy departments in 4 hospitals affiliated with Shanghai University of TCM, namely, Longhua Hospital, Yueyang Hospital of Integrated Traditional Chinese and Western Medicine, Shanghai Traditional Chinese Medicine-Integrated Hospital and Shanghai Municipal Hospital of Traditional Chinese Medicine.

#### Inclusion criteria

Participants will be eligible if they meet all of the following criteria:

1. Individuals aged from 18 to 75 years, regardless of gender
2. Individuals who underwent complete painless endoscopic adenoma resection (excluding hot polypectomy, hot snare polypectomy and argon plasma coagulation) within 2 weeks before recruitment
3. At least 1 but no more than 6 CRAs confirmed by histology (including tubular, tubulovillous, and villous adenoma)
4. Boston Bowel Preparation Scale (BBPS) score ≥7 points
5. The score of SDS in TCM ≥20 points
6. Individuals with adequate organ function
7. Individuals who provide informed consent and can cooperate with long-term follow-up

#### Exclusion criteria

Participants will be excluded if they meet any of the following criteria:

1. The adenoma was not completely removed in previous endoscopy
2. Individuals who regularly take aspirin, non-steroidal anti-inflammatory drugs, cyclo-oxygenase 2 inhibitors, calcium or vitamin D and who receive long-term systemic immunotherapy
3. Pregnant or lactating women
4. Individuals with mental disorders or other conditions who are not able to cooperate
5. Individuals who are unable to take drugs orally, lack physical integrity of the upper gastrointestinal tract, or have malabsorption syndrome and other diseases that investigators consider may interfere with gastrointestinal movement or absorption
6. Individuals who are complicated with acute and chronic diseases, including severe cardiovascular, hepatic and renal diseases, or experience severe postoperative complications
7. Other situations that investigators consider unsuitable for participation

#### Withdrawal/termination criteria

Participants will be withdrawn or terminated from the trial if they meet any of the following criteria:

1. Serious adverse events are reported at any time, and patients are unable to continue
2. Patients who present with serious changes or complications related to the disease during the trial
3. Patients who cannot comply with the instructions and follow-up procedures of this trial, leading to incomplete medical administration records, or patients who took banned drugs or other medications that may interfere with the efficacy and safety evaluation of this study
4. Patients who request to be withdrawn from the trial

Besides, researchers will record the last time of drug intake and collect evaluation items of participants who discontinue or deviate from intervention protocols.

### Interventions

#### Drug intervention

The intervention phase will last 12 months. Both SJZD and placebo are designed as granules with the same weight (1.77g/pack) and packaging. The appearance, texture, smell and taste of placebo granules are the same as SJZD granules but without any therapeutic effect. The granules in one package are dissolved in 200 mL of hot water. Patients are asked to take two packages orally two separate times in 1 day (3.54 g/day). There is no reduction or escalation of drug dose and frequency. SJZD and placebo are provided by Sichuan Neo-green Pharmaceutical Technology Development Co., Ltd. (Sichuan, China). The ingredients of placebo include one-tenth dose of SJZD (Radix ginseng, Rhizoma Atractylodis macrocephalae, Poria and Radix Glycyrrhizae Preparata) and maltodextrin. All the processing and preparation of medications and placebo are qualified. For participants who have severe adverse events (AEs) relevant to this trial, post-trial care will be arranged, and additional pecuniary compensation will be given. For participants who have adverse reactions not related to the drug in this trial, the relevant medical interventions will be implemented based on the comprehensive information of patients, including previous medical histories, symptoms and signs, laboratory, imaging and other examination results.

#### Concomitant treatment regulations

Other medical agents or interventions that may interfere with the evaluation of efficacy and safety in this study will be prohibited during the study. Participants who co-existed with other chronic diseases and need relevant treatments should record concomitant interventions in case report form (CRF).

#### Endoscopic procedures

At every recruiting site, specialists with substantial experience will perform the endoscopic examinations and polypectomies. The clinical practice follows the guideline published by The Japanese Society of Gastroenterology (JSGE) (Tanaka et al., 2021). Adequate bowel cleaning will be required to ensure clear observation of the entire colorectum. All detected adenomas or polyps will be photographed, and standard endoscopic reports will be submitted by the endoscopists and preserved by the principal investigators. Tissue will be collected and stored following the routine clinical care and regulations of the Department of Pathology.

### Outcomes

The primary outcome is the recurrence rate of CRA between the SJZD group and the placebo group at the first year after CRA removal. The number, size and location of adenomas will be recorded. Pathological type will also be recorded if recurrent adenomas meet the criteria for resection. Secondary outcomes include the following: 1) the recurrence rate of CRA at the second year post-polypectomy; 2) the pathological type of adenoma detected at either of the two endoscopies; and 3) the changes in spleen deficiency PRO scores after intervention.

Safety outcomes will comprise physical examinations, vital signs and laboratory examinations at every visit and AEs during the intervention phase. Laboratory indices will include routine blood tests, routine urine tests, routine stool tests, liver function tests, renal function tests and electrocardiograms. In the event of AEs, the onset time, symptoms and signs, duration of event and abnormal laboratory indicators or imaging results will be recorded. The definition and grade assessment of AEs are in accordance with the National Cancer Institute Common Terminology Criteria for Adverse Events (Version 5.0) (SERVICES USDOHAH, 2017).

### Randomization and blinding

Permuted block randomization, which is stratified by recruiting sites, will be used to allocate participants to the SJZD group or placebo group in a 1:1 ratio by the SAS statistical analysis system. All drugs will be sealed in identical opaque envelopes only labeled with a random code number. Participants will be enrolled by clinicians and receive an envelope in sequence. An independent statistician will finish the randomization. Clinicians, nurses, researchers and patients who participate in this trial will all be blinded to the treatment assignment. The random number and treatment assignments will be sealed and reserved by the principal investigators of this study. The blinding will not be unmasked until the trial and data analysis finish or in the event of serious AEs. Emergency letters sealed in opaque envelopes will be sent to each recruiting site. Assignment information and emergent conditions are contained in the letter. If any serious AEs occur, the emergency letter will be opened, and the corresponding patient will be withdrawn from the trial. A report of serious AEs should be submitted to the principal investigators within 24 h. The codes of treatments will be uncovered after the closure of follow-up and the specific intervention relevant to the cords will be uncovered after data analysis.

### Sample size

Based on a previous clinical trial, the effective rate of berberine, a famous Chinese medicine, in the prevention of CRA recurrence was 11% (Chen et al., 2020). The estimated effective rate of SJZD is 20% in this study. Following the 1:1 parallel control principle, SAS statistical analysis software was used to generate the sample size with a double-sided significance level of 0.05 and a power of 80% (α = 0.05, β = 0.2). Assuming that the dropout rate in each group was 10%, a requirement of 240 cases was determined (*n* = 120 in each group).

### Data collection and registration

Data will be recorded in CRF and then entered and stored in REDcap (http://longhua.site/redcap3), a web-based electronic database. All clinical investigators will be trained on how to communicate with patients and how to collect and input information into the electronic database. A total of five visits will be conducted in this trial. Investigators will obtain the informed consent of participants, and then assess the general and demographic information, collect their biomedical and adenomatous information at baseline. They will provide participants with 3 months of drugs/placebo from enrollment to the third visit. For visit 2∼visit 4, patients will be asked to return the empty packages to confirm drug compliance. Telephone calls will be arranged every month to contact patients about their feelings, AEs and adherence to the study. Adenomatous tissue will be collected endoscopically and stored according to the regulations and requirements of the pathological department at visit 4 and visit 5. Original data and collected samples will be preserved at least 5 years after closure of the trial. An independent data monitoring committee (DMC) will be established and will include clinical epidemiologists, data monitors, and statisticians who are not involved in the trial and are independent from sponsors. No competing interest will exist. They will review the documents, check CRFs and relevant data every 6 months to monitor the safety of the study, ensure the authenticity of the profiles and protect confidentiality of participants information. DMC will give suggestions on the study design revision or trial termination.

### Statistical analysis

SAS statistical software will be used to analyze the data. The efficacy and safety of this study will be analyzed by the principle of intention-to-treat (ITT). The missing data will be filled by the method of the last observation carried forward. The measurement data will be expressed as the mean ± standard deviation or median (quartile interval) determined by whether the data are normally distributed and the variance is uniform. χ^2^ tests will be used to compare the primary and secondary endpoints between the SJZD group and the placebo group. The paired *t*-test and independent sample *t*-test will be used for intragroup comparison and intergroup comparison, respectively. Logistic regression analysis will be used for the factors affecting the recurrence rate. Interim analysis will be arranged when 50% of the sample is collected. Sub-group analyses will be determined based on characteristics of participants such as gender, body mass index or previous history of gastrointestinal diseases. *p* < 0.05 is considered statistically significant.

### Trial status

We planed to recruit participants from January 2023 and complete the recruitment in June 2024. The follow-up will finish in September 2025. All clinical data will be locked in November 2025. This protocol was submitted before completion of recruitment.

### Ethics and dissemination

The study conforms to the principles of the Declaration of Helsinki, the Standard Protocol Items: Recommendations for Interventional Trials (SPIRIT) 2013 Statement (Chan et al., 2013) and the Standard Protocol Items for Clinical Trials with Traditional Chinese Medicine: Recommendations, Explanation and Elaboration (SPIRIT-TCM) Extension 2018 Statement (Dai et al., 2019b). The study protocol was approved by the ethics committee of Longhua Hospital Affiliated to Shanghai University of Traditional Chinese Medicine (No. 2022LCSY031) and has been registered in the Chinese Clinical Trial Registry (ChiCTR2200064391). Before the implementation of the study, the protocol was modified twice and approved by the ethics committee. The final protocol version was V2.0. If there will be any changes in study design, ethics committee would be informed promptly. Only participants who sign the informed consent will be enrolled. The informed consent form will be available from the principal investigators on request.

The outcomes of study will be published in a peer-reviewed journal after the study closure. The clinical evidence and experience of trial will be disseminated to participants and the public by conferences and publications. Authorship for the final report will be determined based on the contributions to this study. Professional writers will not be used.

## Discussion

Colorectal cancer is a major health concern globally, with the age-standardized incidence and mortality rate reaching 26.7 and 13.7 per 100,000 population, respectively (Kocarnik et al., 2019). The escalation of colorectal cancers worldwide has prompted clinicians to focus on endoscopic screening, adenoma resection and periodic surveillance after removal to prevent the subsequent development of carcinoma. CRAs, one of the most common precancerous lesions of colorectal cancers, have a relatively high recurrence rate. Previous studies have reported CRAs recurrence rates ranging from 26.6% (Pommergaard et al., 2016) to 51.6% (Higurashi et al., 2016; Chen et al., 2020) after polypectomy, with an observed trend of increased recurrence rates over longer follow-up durations (Gao et al., 2010). Additionally, various factors can influence CRA recurrence rates. While factors such as sex, gene mutations and family history are unmodifiable, endoscopic procedures, diet and chemoprevention agents are modifiable (Hao et al., 2020).

Over the past decades, many studies have explored the potential of certain drugs or nutritional agents in reducing CRA recurrence. A prospective study reported a significant association between higher total vitamin D intake and a reduced risk of conventional adenoma, regardless of the high or low risk of adenomas (Kim et al., 2021). However, this study only recruited young female participants, and extrapolating this result to the general population should be approached cautiously. In a comparative analysis of two RCTs, the Vitamin D/Calcium Polyp Prevention Study (VCPPS, 2004–2013) (Baron et al., 2015) and the Calcium Polyp Prevention Study (CPPS, 1988–1996) (Baron et al., 1999), showed that the protective effect of calcium supplementation on lowering the risk of recurrent CRAs was attenuated by an abnormally high body mass index (Barry et al., 2019). This finding limits the widespread usage of calcium in all patients with CRAs. Recently, a high-quality clinical study reported that berberine, a drug derived from traditional Chinese herbal medicine, effectively reduced the recurrence rate of adenomas after polypectomy (Chen et al., 2020), indicating the promising role of Chinese herbal medicine in the prevention of recurrent CRAs.

Under the guidance of a holistic concept, TCM regards the body as an interconnected system consisting of five organs, referred to as “Zang” in Chinese (not equivalent to the apparatus defined in Western medicine). These organs, including the liver, heart, spleen, lung and kidney (in accordance with “Gan”, “Xin,” “Pi,” “Fei” and “Shen” in Chinese, respectively), work separately but function as a unified entity to maintain the dynamic balance and harmony of the body. Among the five organs, the spleen, located in the central position of the body, not only transforms basic nutritional substances into essence but also regulates energy metabolism. In cases of spleen deficiency, patients often exhibit symptoms similar to those of colorectal diseases, such as anorexia, diarrhea and loose stool, as well as other systemic discomforts, including fatigue, fear of cold and generalized muscle weakness. Based on the TCM principle of “syndrome-disease-prescription,” SDS has been regarded as the main pathogenesis of colorectal cancer (Sun et al., 2016), closely related to the subsequent development of CRAs. As a result, numerous TCM prescriptions have been investigated for their therapeutic effect on SDS patients with colorectal diseases.

SJZD, a renowned prescription in treating SDS over hundreds of years in China, has long been extensively used in gastrointestinal diseases and is favored by clinicians because it can be modified by adding other Chinese herbs under various pathological conditions. To better understand the mechanism of SJZD, the chemical substances of this decoction have been identified (Guan et al., 2018), and the pharmaceutical mechanism of the herbs was also explored with the aid of modern methodology. Studies have found that ginsenoside Rb2, an active substance of ginseng, has an inhibitory effect on the migration, invasion and metastasis of colorectal cancer cells (Dai et al., 2019c). Furthermore, researchers found that non-polysaccharide NPS and polysaccharide S-3, two effective components of SJZD, could stimulate intestinal peristalsis and regulate the immune environment of the intestine in a rat model with SDS (Ma et al., 2021). Other modified decoctions and derivatives of SJZD components have been proven to be effective in treating functional dyspepsia (Lv et al., 2017) and chronic atrophic gastritis (Tian et al., 2019), reducing liver metastasis in a rat model with colon cancer (Zhou et al., 2019), improving gastric movement by restoring the mitochondrial quality control system (Zhang et al., 2022), and inducing apoptosis of colorectal cancer cells (Li et al., 2020). However, the therapeutic effect of SJZD in CRA recurrence is still unknown. Therefore, this trial is designed to explore the efficacy and safety of SJZD in the prevention and treatment of SDS patients after CRA removal.

There are some limitations in this study. First, as there is no reduction or escalation in drug dose, the dose‒response effect will not be compared. Second, the intervention phase is only 12 months, and the follow-up duration is 24 months. A very long-term observation for CRA recurrence will not be completed. Third, the participating sites are all located in Shanghai, and a larger sample size is needed in the future.

In summary, this trial aims to investigate the role of SJZD in patients with SDS after CRA polypectomy. Under the guidance of TCM, we hope to accumulate more experience for clinical practice and provide high-quality evidence of herbal medicine in coping with colorectal disease.
